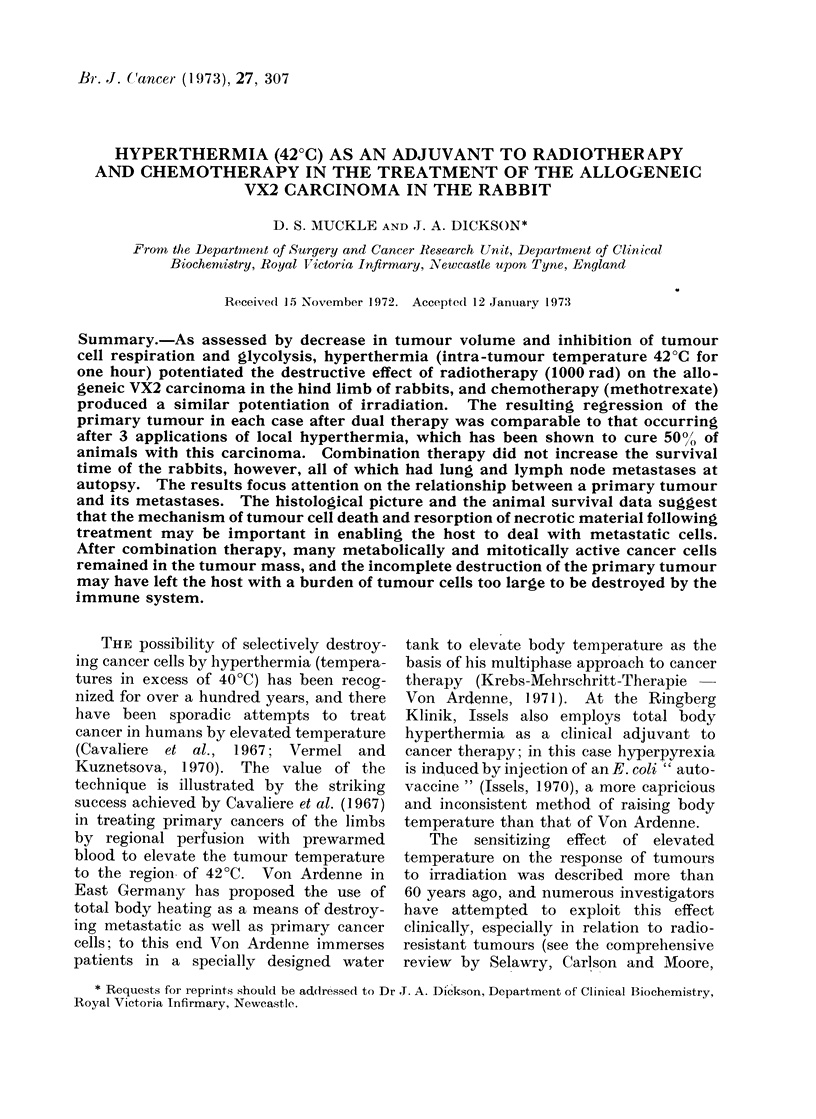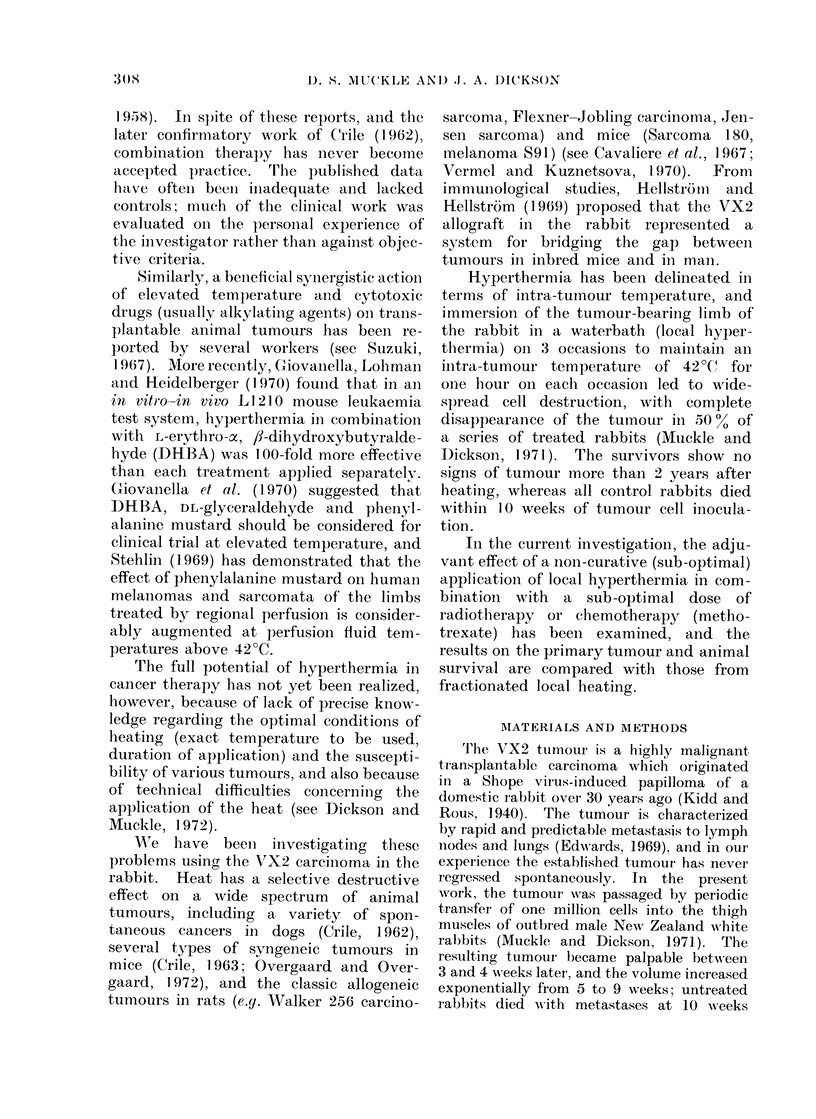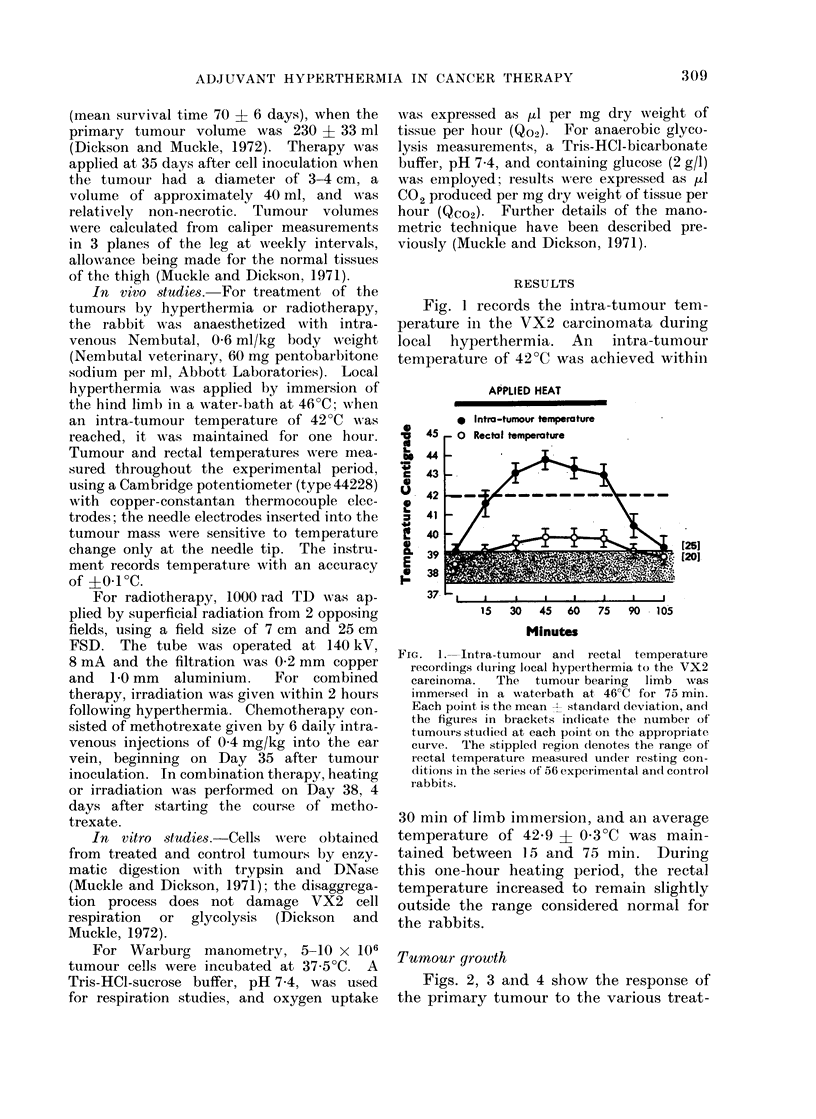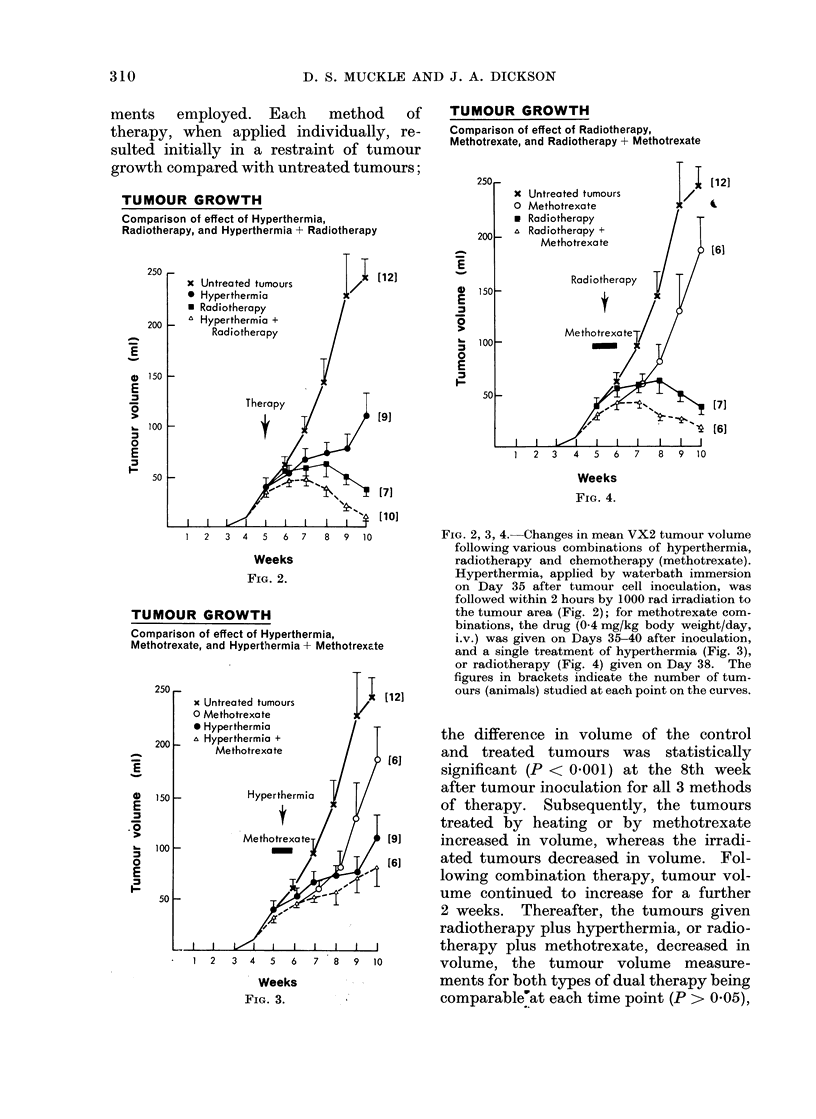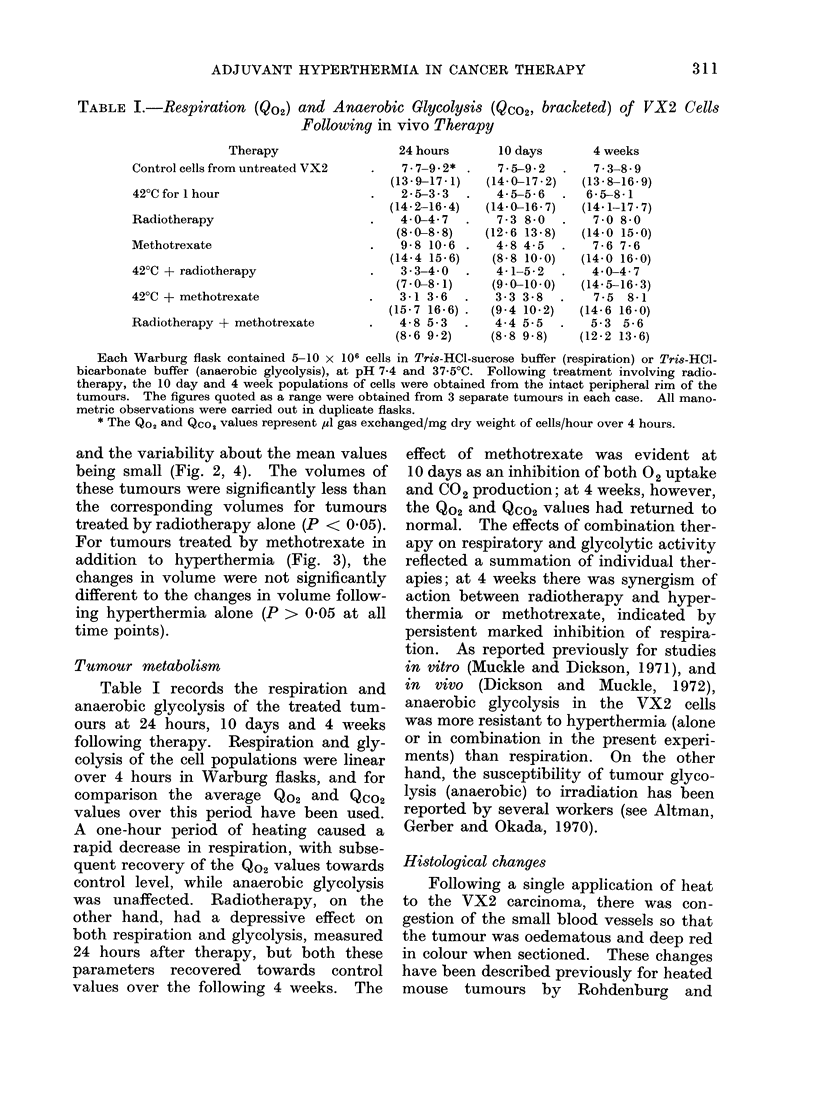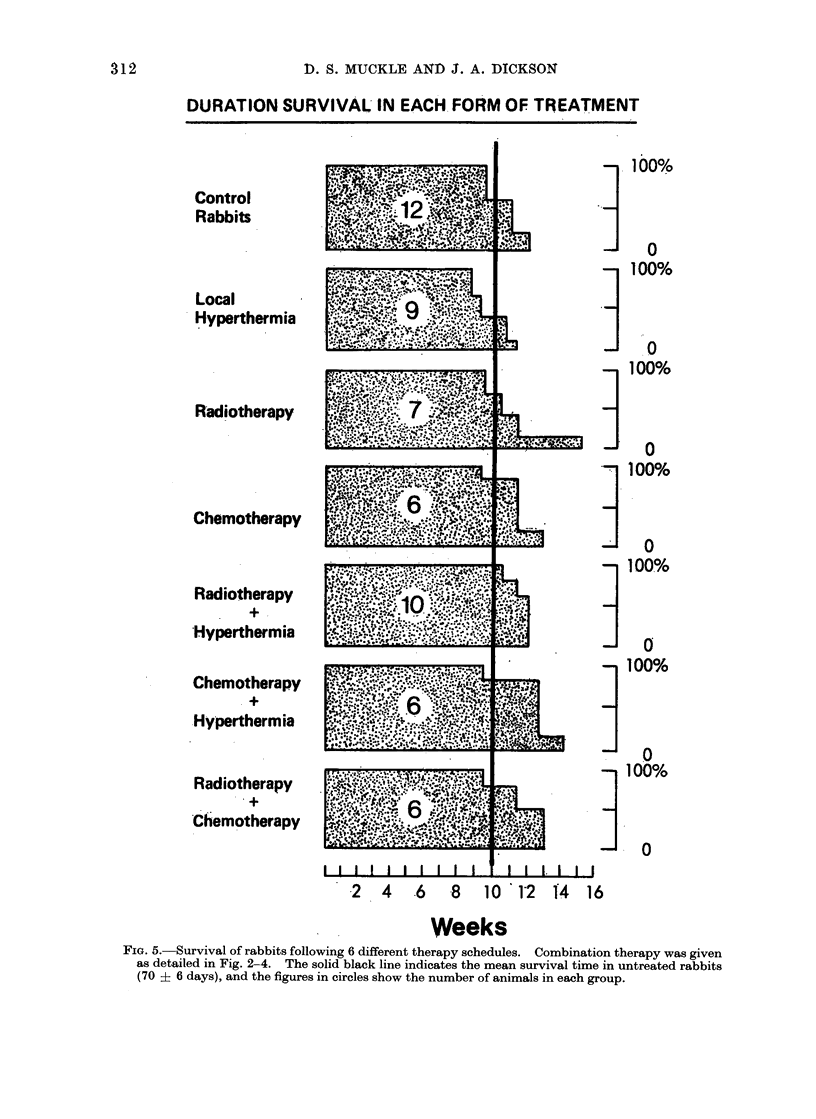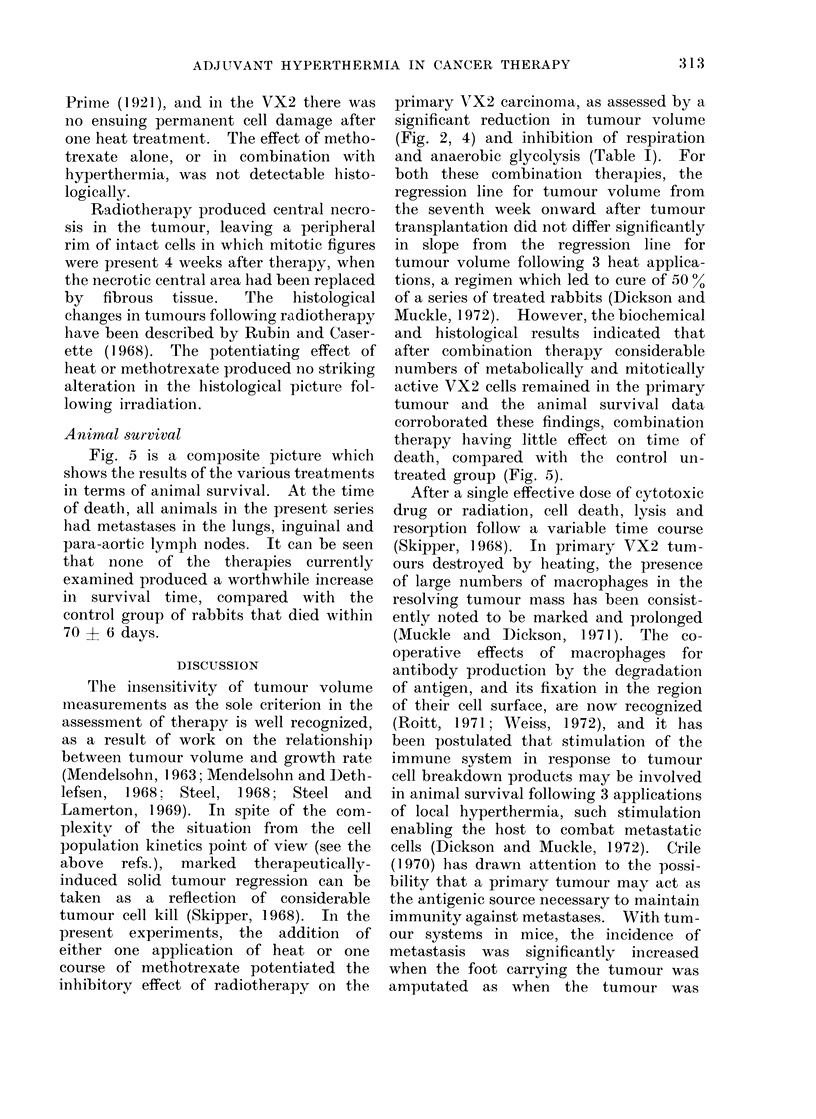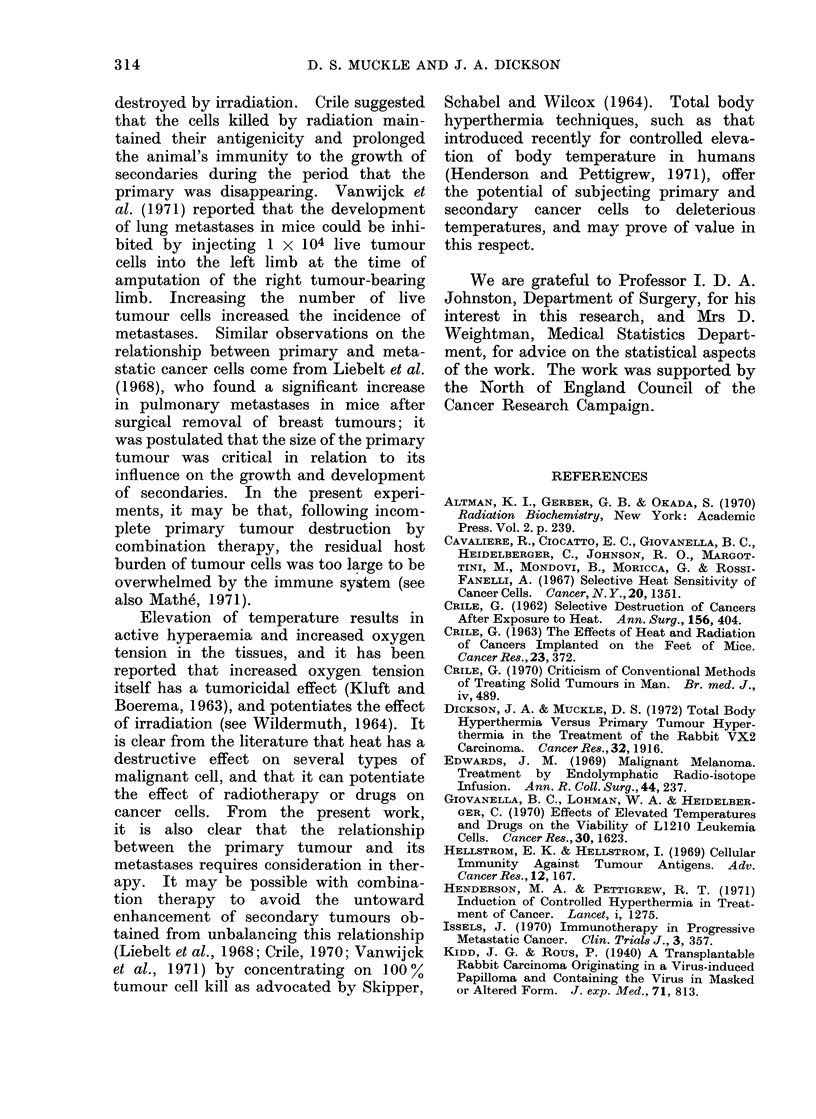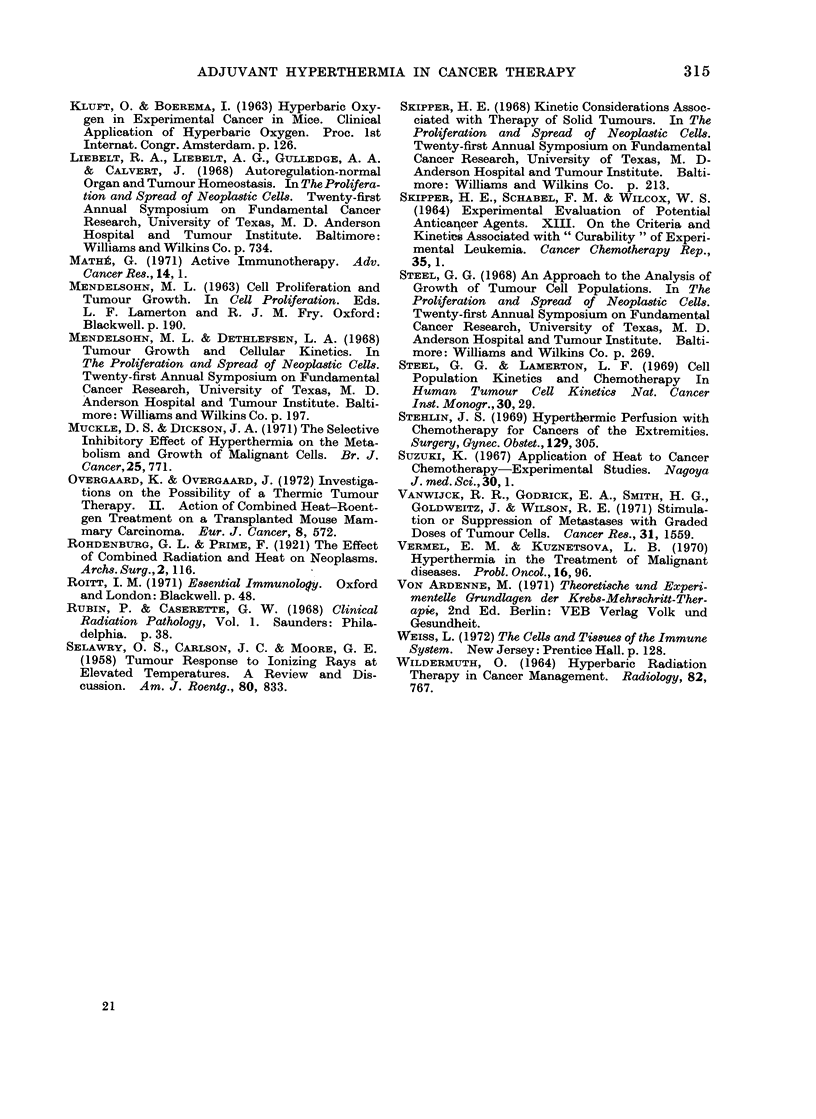# Hyperthermia (42°C) as an Adjuvant to Radiotherapy and Chemotherapy in the Treatment of the Allogeneic VX2 Carcinoma in the Rabbit

**DOI:** 10.1038/bjc.1973.37

**Published:** 1973-04

**Authors:** D. S. Muckle, J. A. Dickson

## Abstract

As assessed by decrease in tumour volume and inhibition of tumour cell respiration and glycolysis, hyperthermia (intra-tumour temperature 42°C for one hour) potentiated the destructive effect of radiotherapy (1000 rad) on the allogeneic VX2 carcinoma in the hind limb of rabbits, and chemotherapy (methotrexate) produced a similar potentiation of irradiation. The resulting regression of the primary tumour in each case after dual therapy was comparable to that occurring after 3 applications of local hyperthermia, which has been shown to cure 50% of animals with this carcinoma. Combination therapy did not increase the survival time of the rabbits, however, all of which had lung and lymph node metastases at autopsy. The results focus attention on the relationship between a primary tumour and its metastases. The histological picture and the animal survival data suggest that the mechanism of tumour cell death and resorption of necrotic material following treatment may be important in enabling the host to deal with metastatic cells. After combination therapy, many metabolically and mitotically active cancer cells remained in the tumour mass, and the incomplete destruction of the primary tumour may have left the host with a burden of tumour cells too large to be destroyed by the immune system.


Br. J. Cancer (1973), 27, 307

### HYPERTHERMIA (42°C) AS AN ADJUVANT TO RADIOTHERAPY AND CHEMOTHERAPY IN THE TREATMENT OF THE ALLOGENEIC VX2 CARCINOMA IN THE RABBIT

D. S. MUCKLE AND J. A. DICKSON*

*From the Department of Surgery and Cancer Research Unit, Department of Clinical Biochemistry, Royal Victoria Infirmary, Newcastle upon Tyne, England*

Received 15 November 1972. Accepted 12 January 1973

**Summary.—As assessed by decrease in tumour volume and inhibition of tumour cell respiration and glycolysis, hyperthermia (intra-tumour temperature 42°C for one hour) potentiated the destructive effect of radiotherapy (1000 rad) on the allogeneic VX2 carcinoma in the hind limb of rabbits, and chemotherapy (methotrexate) produced a similar potentiation of irradiation. The resulting regression of the primary tumour in each case after dual therapy was comparable to that occurring after 3 applications of local hyperthermia, which has been shown to cure 50% of animals with this carcinoma. Combination therapy did not increase the survival time of the rabbits, however, all of which had lung and lymph node metastases at autopsy. The results focus attention on the relationship between a primary tumour and its metastases. The histological picture and the animal survival data suggest that the mechanism of tumour cell death and resorption of necrotic material following treatment may be important in enabling the host to deal with metastatic cells. After combination therapy, many metabolically and mitotically active cancer cells remained in the tumour mass, and the incomplete destruction of the primary tumour may have left the host with a burden of tumour cells too large to be destroyed by the immune system.**

THE possibility of selectively destroying cancer cells by hyperthermia (temperatures in excess of 40°C) has been recognized for over a hundred years, and there have been sporadic attempts to treat cancer in humans by elevated temperature (Cavaliere *et al.*, 1967; Vermel and Kuznetsova, 1970). The value of the technique is illustrated by the striking success achieved by Cavaliere *et al.* (1967) in treating primary cancers of the limbs by regional perfusion with prewarmed blood to elevate the tumour temperature to the region of 42°C. Von Ardenne in East Germany has proposed the use of total body heating as a means of destroying metastatic as well as primary cancer cells; to this end Von Ardenne immerses patients in a specially designed water tank to elevate body temperature as the basis of his multiphase approach to cancer therapy (Krebs-Mehrschritt-Therapie — Von Ardenne, 1971). At the Ringberg Klinik, Issels also employs total body hyperthermia as a clinical adjuvant to cancer therapy; in this case hyperpyrexia is induced by injection of an *E. coli* " autovaccine " (Issels, 1970), a more capricious and inconsistent method of raising body temperature than that of Von Ardenne.

The sensitizing effect of elevated temperature on the response of tumours to irradiation was described more than 60 years ago, and numerous investigators have attempted to exploit this effect clinically, especially in relation to radioresistant tumours (see the comprehensive review by Selawry, Carlson and Moore,

* Requests for reprints should be addressed to Dr J. A. Dickson, Department of Clinical Biochemistry, Royal Victoria Infirmary, Newcastle.

1958). In spite of these reports, and the later confirmatory work of Crile (1962), combination therapy has never become accepted practice. The published data have often been inadequate and lacked controls; much of the clinical work was evaluated on the personal experience of the investigator rather than against objective criteria.

Similarly, a beneficial synergistic action of elevated temperature and cytotoxic drugs (usually alkylating agents) on transplantable animal tumours has been reported by several workers (see Suzuki, 1967). More recently, Giovanella, Lohman and Heidelberger (1970) found that in an *in vitro–in vivo* L1210 mouse leukaemia test system, hyperthermia in combination with L-erythro-$\alpha$, $\beta$-dihydroxybutyraldehyde (DHBA) was 100-fold more effective than each treatment applied separately. Giovanella *et al.* (1970) suggested that DHBA, DL-glyceraldehyde and phenylalanine mustard should be considered for clinical trial at elevated temperature, and Stehlin (1969) has demonstrated that the effect of phenylalanine mustard on human melanomas and sarcomata of the limbs treated by regional perfusion is considerably augmented at perfusion fluid temperatures above 42°C.

The full potential of hyperthermia in cancer therapy has not yet been realized, however, because of lack of precise knowledge regarding the optimal conditions of heating (exact temperature to be used, duration of application) and the susceptibility of various tumours, and also because of technical difficulties concerning the application of the heat (see Dickson and Muckle, 1972).

We have been investigating these problems using the VX2 carcinoma in the rabbit. Heat has a selective destructive effect on a wide spectrum of animal tumours, including a variety of spontaneous cancers in dogs (Crile, 1962), several types of syngeneic tumours in mice (Crile, 1963; Overgaard and Overgaard, 1972), and the classic allogeneic tumours in rats (*e.g.* Walker 256 carcinosarcoma, Flexner–Jobling carcinoma, Jensen sarcoma) and mice (Sarcoma 180, melanoma S91) (see Cavaliere *et al.*, 1967; Vermel and Kuznetsova, 1970). From immunological studies, Hellström and Hellström (1969) proposed that the VX2 allograft in the rabbit represented a system for bridging the gap between tumours in inbred mice and in man.

Hyperthermia has been delineated in terms of intra-tumour temperature, and immersion of the tumour-bearing limb of the rabbit in a waterbath (local hyperthermia) on 3 occasions to maintain an intra-tumour temperature of 42°C for one hour on each occasion led to widespread cell destruction, with complete disappearance of the tumour in 50% of a series of treated rabbits (Muckle and Dickson, 1971). The survivors show no signs of tumour more than 2 years after heating, whereas all control rabbits died within 10 weeks of tumour cell inoculation.

In the current investigation, the adjuvant effect of a non-curative (sub-optimal) application of local hyperthermia in combination with a sub-optimal dose of radiotherapy or chemotherapy (methotrexate) has been examined, and the results on the primary tumour and animal survival are compared with those from fractionated local heating.

### MATERIALS AND METHODS

The VX2 tumour is a highly malignant transplantable carcinoma which originated in a Shope virus-induced papilloma of a domestic rabbit over 30 years ago (Kidd and Rous, 1940). The tumour is characterized by rapid and predictable metastasis to lymph nodes and lungs (Edwards, 1969), and in our experience the established tumour has never regressed spontaneously. In the present work, the tumour was passaged by periodic transfer of one million cells into the thigh muscles of outbred male New Zealand white rabbits (Muckle and Dickson, 1971). The resulting tumour became palpable between 3 and 4 weeks later, and the volume increased exponentially from 5 to 9 weeks; untreated rabbits died with metastases at 10 weeks

(mean survival time 70 ± 6 days), when the primary tumour volume was 230 ± 33 ml (Dickson and Muckle, 1972). Therapy was applied at 35 days after cell inoculation when the tumour had a diameter of 3–4 cm, a volume of approximately 40 ml, and was relatively non-necrotic. Tumour volumes were calculated from caliper measurements in 3 planes of the leg at weekly intervals, allowance being made for the normal tissues of the thigh (Muckle and Dickson, 1971).

*In vivo studies*.—For treatment of the tumours by hyperthermia or radiotherapy, the rabbit was anaesthetized with intravenous Nembutal, 0·6 ml/kg body weight (Nembutal veterinary, 60 mg pentobarbitone sodium per ml, Abbott Laboratories). Local hyperthermia was applied by immersion of the hind limb in a water-bath at 46°C; when an intra-tumour temperature of 42°C was reached, it was maintained for one hour. Tumour and rectal temperatures were measured throughout the experimental period, using a Cambridge potentiometer (type 44228) with copper-constantan thermocouple electrodes; the needle electrodes inserted into the tumour mass were sensitive to temperature change only at the needle tip. The instrument records temperature with an accuracy of ±0·1°C.

For radiotherapy, 1000 rad TD was applied by superficial radiation from 2 opposing fields, using a field size of 7 cm and 25 cm FSD. The tube was operated at 140 kV, 8 mA and the filtration was 0·2 mm copper and 1·0 mm aluminium. For combined therapy, irradiation was given within 2 hours following hyperthermia. Chemotherapy consisted of methotrexate given by 6 daily intravenous injections of 0·4 mg/kg into the ear vein, beginning on Day 35 after tumour inoculation. In combination therapy, heating or irradiation was performed on Day 38, 4 days after starting the course of methotrexate.

*In vitro studies*.—Cells were obtained from treated and control tumours by enzymatic digestion with trypsin and DNase (Muckle and Dickson, 1971); the disaggregation process does not damage VX2 cell respiration or glycolysis (Dickson and Muckle, 1972).

For Warburg manometry, 5–10 × $10^6$ tumour cells were incubated at 37·5°C. A Tris-HCl-sucrose buffer, pH 7·4, was used for respiration studies, and oxygen uptake was expressed as $\mu$l per mg dry weight of tissue per hour ($Q_{O_2}$). For anaerobic glycolysis measurements, a Tris-HCl-bicarbonate buffer, pH 7·4, and containing glucose (2 g/l) was employed; results were expressed as $\mu$l $CO_2$ produced per mg dry weight of tissue per hour ($Q_{CO_2}$). Further details of the manometric technique have been described previously (Muckle and Dickson, 1971).

RESULTS

Fig. 1 records the intra-tumour temperature in the VX2 carcinomata during local hyperthermia. An intra-tumour temperature of 42°C was achieved within

FIG. 1.—Intra-tumour and rectal temperature recordings during local hyperthermia to the VX2 carcinoma. The tumour bearing limb was immersed in a waterbath at 46°C for 75 min. Each point is the mean ± standard deviation, and the figures in brackets indicate the number of tumours studied at each point on the appropriate curve. The stippled region denotes the range of rectal temperature measured under resting conditions in the series of 56 experimental and control rabbits.

30 min of limb immersion, and an average temperature of 42·9 ± 0·3°C was maintained between 15 and 75 min. During this one-hour heating period, the rectal temperature increased to remain slightly outside the range considered normal for the rabbits.

*Tumour growth*

Figs. 2, 3 and 4 show the response of the primary tumour to the various treatments employed. Each method of therapy, when applied individually, resulted initially in a restraint of tumour growth compared with untreated tumours;

**TUMOUR GROWTH**

**Comparison of effect of Hyperthermia, Radiotherapy, and Hyperthermia + Radiotherapy**

FIG. 2.

**TUMOUR GROWTH**

**Comparison of effect of Hyperthermia, Methotrexate, and Hyperthermia + Methotrexate**

FIG. 3.

**TUMOUR GROWTH**

**Comparison of effect of Radiotherapy, Methotrexate, and Radiotherapy + Methotrexate**

FIG. 4.

FIG. 2, 3, 4.—Changes in mean VX2 tumour volume following various combinations of hyperthermia, radiotherapy and chemotherapy (methotrexate). Hyperthermia, applied by waterbath immersion on Day 35 after tumour cell inoculation, was followed within 2 hours by 1000 rad irradiation to the tumour area (Fig. 2); for methotrexate combinations, the drug (0·4 mg/kg body weight/day, i.v.) was given on Days 35–40 after inoculation, and a single treatment of hyperthermia (Fig. 3), or radiotherapy (Fig. 4) given on Day 38. The figures in brackets indicate the number of tumours (animals) studied at each point on the curves.

the difference in volume of the control and treated tumours was statistically significant ($P < 0{\cdot}001$) at the 8th week after tumour inoculation for all 3 methods of therapy. Subsequently, the tumours treated by heating or by methotrexate increased in volume, whereas the irradiated tumours decreased in volume. Following combination therapy, tumour volume continued to increase for a further 2 weeks. Thereafter, the tumours given radiotherapy plus hyperthermia, or radiotherapy plus methotrexate, decreased in volume, the tumour volume measurements for both types of dual therapy being comparable at each time point ($P > 0{\cdot}05$),

TABLE I.—*Respiration* ($Q_{O_2}$) *and Anaerobic Glycolysis* ($Q_{CO_2}$, *bracketed*) *of VX2 Cells Following* in vivo *Therapy*

| Therapy | 24 hours | 10 days | 4 weeks |
|---|---|---|---|
| Control cells from untreated VX2 | 7·7–9·2* (13·9–17·1) | 7·5–9·2 (14·0–17·2) | 7·3–8·9 (13·8–16·9) |
| 42°C for 1 hour | 2·5–3·3 (14·2–16·4) | 4·5–5·6 (14·0–16·7) | 6·5–8·1 (14·1–17·7) |
| Radiotherapy | 4·0–4·7 (8·0–8·8) | 7·3 8·0 (12·6 13·8) | 7·0 8·0 (14·0 15·0) |
| Methotrexate | 9·8 10·6 (14·4 15·6) | 4·8 4·5 (8·8 10·0) | 7·6 7·6 (14·0 16·0) |
| 42°C + radiotherapy | 3·3–4·0 (7·0–8·1) | 4·1–5·2 (9·0–10·0) | 4·0–4·7 (14·5–16·3) |
| 42°C + methotrexate | 3·1 3·6 (15·7 16·6) | 3·3 3·8 (9·4 10·2) | 7·5 8·1 (14·6 16·0) |
| Radiotherapy + methotrexate | 4·8 5·3 (8·6 9·2) | 4·4 5·5 (8·8 9·8) | 5·3 5·6 (12·2 13·6) |

Each Warburg flask contained 5–10 × $10^6$ cells in *Tris*-HCl-sucrose buffer (respiration) or *Tris*-HCl-bicarbonate buffer (anaerobic glycolysis), at pH 7·4 and 37·5°C. Following treatment involving radiotherapy, the 10 day and 4 week populations of cells were obtained from the intact peripheral rim of the tumours. The figures quoted as a range were obtained from 3 separate tumours in each case. All manometric observations were carried out in duplicate flasks.

* The $Q_{O_2}$ and $Q_{CO_2}$ values represent $\mu$l gas exchanged/mg dry weight of cells/hour over 4 hours.

and the variability about the mean values being small (Fig. 2, 4). The volumes of these tumours were significantly less than the corresponding volumes for tumours treated by radiotherapy alone ($P < 0{\cdot}05$). For tumours treated by methotrexate in addition to hyperthermia (Fig. 3), the changes in volume were not significantly different to the changes in volume following hyperthermia alone ($P > 0{\cdot}05$ at all time points).

*Tumour metabolism*

Table I records the respiration and anaerobic glycolysis of the treated tumours at 24 hours, 10 days and 4 weeks following therapy. Respiration and glycolysis of the cell populations were linear over 4 hours in Warburg flasks, and for comparison the average $Q_{O_2}$ and $Q_{CO_2}$ values over this period have been used. A one-hour period of heating caused a rapid decrease in respiration, with subsequent recovery of the $Q_{O_2}$ values towards control level, while anaerobic glycolysis was unaffected. Radiotherapy, on the other hand, had a depressive effect on both respiration and glycolysis, measured 24 hours after therapy, but both these parameters recovered towards control values over the following 4 weeks. The effect of methotrexate was evident at 10 days as an inhibition of both $O_2$ uptake and $CO_2$ production; at 4 weeks, however, the $Q_{O_2}$ and $Q_{CO_2}$ values had returned to normal. The effects of combination therapy on respiratory and glycolytic activity reflected a summation of individual therapies; at 4 weeks there was synergism of action between radiotherapy and hyperthermia or methotrexate, indicated by persistent marked inhibition of respiration. As reported previously for studies *in vitro* (Muckle and Dickson, 1971), and *in vivo* (Dickson and Muckle, 1972), anaerobic glycolysis in the VX2 cells was more resistant to hyperthermia (alone or in combination in the present experiments) than respiration. On the other hand, the susceptibility of tumour glycolysis (anaerobic) to irradiation has been reported by several workers (see Altman, Gerber and Okada, 1970).

*Histological changes*

Following a single application of heat to the VX2 carcinoma, there was congestion of the small blood vessels so that the tumour was oedematous and deep red in colour when sectioned. These changes have been described previously for heated mouse tumours by Rohdenburg and

FIG. 5.—Survival of rabbits following 6 different therapy schedules. Combination therapy was given as detailed in Fig. 2–4. The solid black line indicates the mean survival time in untreated rabbits (70 ± 6 days), and the figures in circles show the number of animals in each group.

Prime (1921), and in the VX2 there was no ensuing permanent cell damage after one heat treatment. The effect of methotrexate alone, or in combination with hyperthermia, was not detectable histologically.

Radiotherapy produced central necrosis in the tumour, leaving a peripheral rim of intact cells in which mitotic figures were present 4 weeks after therapy, when the necrotic central area had been replaced by fibrous tissue. The histological changes in tumours following radiotherapy have been described by Rubin and Caserette (1968). The potentiating effect of heat or methotrexate produced no striking alteration in the histological picture following irradiation.

*Animal survival*

Fig. 5 is a composite picture which shows the results of the various treatments in terms of animal survival. At the time of death, all animals in the present series had metastases in the lungs, inguinal and para-aortic lymph nodes. It can be seen that none of the therapies currently examined produced a worthwhile increase in survival time, compared with the control group of rabbits that died within $70 \pm 6$ days.

DISCUSSION

The insensitivity of tumour volume measurements as the sole criterion in the assessment of therapy is well recognized, as a result of work on the relationship between tumour volume and growth rate (Mendelsohn, 1963; Mendelsohn and Dethlefsen, 1968; Steel, 1968; Steel and Lamerton, 1969). In spite of the complexity of the situation from the cell population kinetics point of view (see the above refs.), marked therapeutically-induced solid tumour regression can be taken as a reflection of considerable tumour cell kill (Skipper, 1968). In the present experiments, the addition of either one application of heat or one course of methotrexate potentiated the inhibitory effect of radiotherapy on the primary VX2 carcinoma, as assessed by a significant reduction in tumour volume (Fig. 2, 4) and inhibition of respiration and anaerobic glycolysis (Table I). For both these combination therapies, the regression line for tumour volume from the seventh week onward after tumour transplantation did not differ significantly in slope from the regression line for tumour volume following 3 heat applications, a regimen which led to cure of 50% of a series of treated rabbits (Dickson and Muckle, 1972). However, the biochemical and histological results indicated that after combination therapy considerable numbers of metabolically and mitotically active VX2 cells remained in the primary tumour and the animal survival data corroborated these findings, combination therapy having little effect on time of death, compared with the control untreated group (Fig. 5).

After a single effective dose of cytotoxic drug or radiation, cell death, lysis and resorption follow a variable time course (Skipper, 1968). In primary VX2 tumours destroyed by heating, the presence of large numbers of macrophages in the resolving tumour mass has been consistently noted to be marked and prolonged (Muckle and Dickson, 1971). The cooperative effects of macrophages for antibody production by the degradation of antigen, and its fixation in the region of their cell surface, are now recognized (Roitt, 1971; Weiss, 1972), and it has been postulated that stimulation of the immune system in response to tumour cell breakdown products may be involved in animal survival following 3 applications of local hyperthermia, such stimulation enabling the host to combat metastatic cells (Dickson and Muckle, 1972). Crile (1970) has drawn attention to the possibility that a primary tumour may act as the antigenic source necessary to maintain immunity against metastases. With tumour systems in mice, the incidence of metastasis was significantly increased when the foot carrying the tumour was amputated as when the tumour was destroyed by irradiation. Crile suggested that the cells killed by radiation maintained their antigenicity and prolonged the animal's immunity to the growth of secondaries during the period that the primary was disappearing. Vanwijck *et al.* (1971) reported that the development of lung metastases in mice could be inhibited by injecting $1 \times 10^4$ live tumour cells into the left limb at the time of amputation of the right tumour-bearing limb. Increasing the number of live tumour cells increased the incidence of metastases. Similar observations on the relationship between primary and metastatic cancer cells come from Liebelt *et al.* (1968), who found a significant increase in pulmonary metastases in mice after surgical removal of breast tumours; it was postulated that the size of the primary tumour was critical in relation to its influence on the growth and development of secondaries. In the present experiments, it may be that, following incomplete primary tumour destruction by combination therapy, the residual host burden of tumour cells was too large to be overwhelmed by the immune system (see also Mathé, 1971).

Elevation of temperature results in active hyperaemia and increased oxygen tension in the tissues, and it has been reported that increased oxygen tension itself has a tumoricidal effect (Kluft and Boerema, 1963), and potentiates the effect of irradiation (see Wildermuth, 1964). It is clear from the literature that heat has a destructive effect on several types of malignant cell, and that it can potentiate the effect of radiotherapy or drugs on cancer cells. From the present work, it is also clear that the relationship between the primary tumour and its metastases requires consideration in therapy. It may be possible with combination therapy to avoid the untoward enhancement of secondary tumours obtained from unbalancing this relationship (Liebelt *et al.*, 1968; Crile, 1970; Vanwijck *et al.*, 1971) by concentrating on 100% tumour cell kill as advocated by Skipper, Schabel and Wilcox (1964). Total body hyperthermia techniques, such as that introduced recently for controlled elevation of body temperature in humans (Henderson and Pettigrew, 1971), offer the potential of subjecting primary and secondary cancer cells to deleterious temperatures, and may prove of value in this respect.

We are grateful to Professor I. D. A. Johnston, Department of Surgery, for his interest in this research, and Mrs D. Weightman, Medical Statistics Department, for advice on the statistical aspects of the work. The work was supported by the North of England Council of the Cancer Research Campaign.

### REFERENCES

ALTMAN, K. I., GERBER, G. B. & OKADA, S. (1970) *Radiation Biochemistry*, New York: Academic Press. Vol. 2. p. 239.

CAVALIERE, R., CIOCATTO, E. C., GIOVANELLA, B. C., HEIDELBERGER, C., JOHNSON, R. O., MARGOTTINI, M., MONDOVI, B., MORICCA, G. & ROSSI-FANELLI, A. (1967) Selective Heat Sensitivity of Cancer Cells. *Cancer, N.Y.*, **20**, 1351.

CRILE, G. (1962) Selective Destruction of Cancers After Exposure to Heat. *Ann. Surg.*, **156**, 404.

CRILE, G. (1963) The Effects of Heat and Radiation of Cancers Implanted on the Feet of Mice. *Cancer Res.*, **23**, 372.

CRILE, G. (1970) Criticism of Conventional Methods of Treating Solid Tumours in Man. *Br. med. J.*, iv, 489.

DICKSON, J. A. & MUCKLE, D. S. (1972) Total Body Hyperthermia Versus Primary Tumour Hyperthermia in the Treatment of the Rabbit VX2 Carcinoma. *Cancer Res.*, **32**, 1916.

EDWARDS, J. M. (1969) Malignant Melanoma. Treatment by Endolymphatic Radio-isotope Infusion. *Ann. R. Coll. Surg.*, **44**, 237.

GIOVANELLA, B. C., LOHMAN, W. A. & HEIDELBERGER, C. (1970) Effects of Elevated Temperatures and Drugs on the Viability of L1210 Leukemia Cells. *Cancer Res.*, **30**, 1623.

HELLSTROM, E. K. & HELLSTROM, I. (1969) Cellular Immunity Against Tumour Antigens. *Adv. Cancer Res.*, **12**, 167.

HENDERSON, M. A. & PETTIGREW, R. T. (1971) Induction of Controlled Hyperthermia in Treatment of Cancer. *Lancet*, i, 1275.

ISSELS, J. (1970) Immunotherapy in Progressive Metastatic Cancer. *Clin. Trials J.*, **3**, 357.

KIDD, J. G. & ROUS, P. (1940) A Transplantable Rabbit Carcinoma Originating in a Virus-induced Papilloma and Containing the Virus in Masked or Altered Form. *J. exp. Med.*, **71**, 813.

KLUFT, O. & BOEREMA, I. (1963) Hyperbaric Oxygen in Experimental Cancer in Mice. Clinical Application of Hyperbaric Oxygen. Proc. 1st Internat. Congr. Amsterdam. p. 126.

LIEBELT, R. A., LIEBELT, A. G., GULLEDGE, A. A. & CALVERT, J. (1968) Autoregulation-normal Organ and Tumour Homeostasis. In *The Proliferation and Spread of Neoplastic Cells.* Twenty-first Annual Symposium on Fundamental Cancer Research, University of Texas, M. D. Anderson Hospital and Tumour Institute. Baltimore: Williams and Wilkins Co. p. 734.

MATHÉ, G. (1971) Active Immunotherapy. *Adv. Cancer Res.*, **14**, 1.

MENDELSOHN, M. L. (1963) Cell Proliferation and Tumour Growth. In *Cell Proliferation.* Eds. L. F. Lamerton and R. J. M. Fry. Oxford: Blackwell. p. 190.

MENDELSOHN, M. L. & DETHLEFSEN, L. A. (1968) Tumour Growth and Cellular Kinetics. In *The Proliferation and Spread of Neoplastic Cells.* Twenty-first Annual Symposium on Fundamental Cancer Research, University of Texas, M. D. Anderson Hospital and Tumour Institute. Baltimore: Williams and Wilkins Co. p. 197.

MUCKLE, D. S. & DICKSON, J. A. (1971) The Selective Inhibitory Effect of Hyperthermia on the Metabolism and Growth of Malignant Cells. *Br. J. Cancer*, **25**, 771.

OVERGAARD, K. & OVERGAARD, J. (1972) Investigations on the Possibility of a Thermic Tumour Therapy. II. Action of Combined Heat–Roentgen Treatment on a Transplanted Mouse Mammary Carcinoma. *Eur. J. Cancer*, **8**, 572.

ROHDENBURG, G. L. & PRIME, F. (1921) The Effect of Combined Radiation and Heat on Neoplasms. *Archs. Surg.*, **2**, 116.

ROITT, I. M. (1971) *Essential Immunology.* Oxford and London: Blackwell. p. 48.

RUBIN, P. & CASERETTE, G. W. (1968) *Clinical Radiation Pathology*, Vol. 1. Saunders: Philadelphia. p. 38.

SELAWRY, O. S., CARLSON, J. C. & MOORE, G. E. (1958) Tumour Response to Ionizing Rays at Elevated Temperatures. A Review and Discussion. *Am. J. Roentg.*, **80**, 833.

SKIPPER, H. E. (1968) Kinetic Considerations Associated with Therapy of Solid Tumours. In *The Proliferation and Spread of Neoplastic Cells.* Twenty-first Annual Symposium on Fundamental Cancer Research, University of Texas, M. D-Anderson Hospital and Tumour Institute. Baltimore: Williams and Wilkins Co. p. 213.

SKIPPER, H. E., SCHABEL, F. M. & WILCOX, W. S. (1964) Experimental Evaluation of Potential Anticancer Agents. XIII. On the Criteria and Kinetics Associated with "Curability" of Experimental Leukemia. *Cancer Chemotherapy Rep.*, **35**, 1.

STEEL, G. G. (1968) An Approach to the Analysis of Growth of Tumour Cell Populations. In *The Proliferation and Spread of Neoplastic Cells.* Twenty-first Annual Symposium on Fundamental Cancer Research, University of Texas, M. D. Anderson Hospital and Tumour Institute. Baltimore: Williams and Wilkins Co. p. 269.

STEEL, G. G. & LAMERTON, L. F. (1969) Cell Population Kinetics and Chemotherapy In *Human Tumour Cell Kinetics Nat. Cancer Inst. Monogr.*, **30**, 29.

STEHLIN, J. S. (1969) Hyperthermic Perfusion with Chemotherapy for Cancers of the Extremities. *Surgery, Gynec. Obstet.*, **129**, 305.

SUZUKI, K. (1967) Application of Heat to Cancer Chemotherapy—Experimental Studies. *Nagoya J. med. Sci.*, **30**, 1.

VANWIJCK, R. R., GODRICK, E. A., SMITH, H. G., GOLDWEITZ, J. & WILSON, R. E. (1971) Stimulation or Suppression of Metastases with Graded Doses of Tumour Cells. *Cancer Res.*, **31**, 1559.

VERMEL, E. M. & KUZNETSOVA, L. B. (1970) Hyperthermia in the Treatment of Malignant diseases. *Probl. Oncol.*, **16**, 96.

VON ARDENNE, M. (1971) *Theoretische und Experimentelle Grundlagen der Krebs-Mehrschritt-Therapie*, 2nd Ed. Berlin: VEB Verlag Volk und Gesundheit.

WEISS, L. (1972) *The Cells and Tissues of the Immune System.* New Jersey: Prentice Hall. p. 128.

WILDERMUTH, O. (1964) Hyperbaric Radiation Therapy in Cancer Management. *Radiology*, **82**, 767.